# Niacin and Butyrate: Nutraceuticals Targeting Dysbiosis and Intestinal Permeability in Parkinson’s Disease

**DOI:** 10.3390/nu13010028

**Published:** 2020-12-23

**Authors:** Tennekoon B. Karunaratne, Chijioke Okereke, Marissa Seamon, Sharad Purohit, Chandramohan Wakade, Amol Sharma

**Affiliations:** 1 Digestive Health Clinical Research Center, Division of Gastroenterology/Hepatology, Medical College of Georgia, Augusta University, 1120, 15th St, Augusta, GA 30912, USA; tkarunaratne@augusta.edu (T.B.K.); cokereke@augusta.edu (C.O.); 2 Center for Biotechnology and Genomic Medicine, Medical College of Georgia, Augusta University, 1120, 15th St, Augusta, GA 30912, USA; mseamon@augusta.edu (M.S.); spurohit@augusta.edu (S.P.); cwakade@augusta.edu (C.W.); 3 Department of Neuroscience, Medical College of Georgia, Augusta University, 1120, 15th St, Augusta, GA 30912, USA; 4 Department of Obstetrics and Gynecology, Medical College of Georgia, Augusta University, 1120, 15th St, Augusta, GA 30912, USA; 5 Department of Undergraduate Health Professionals, College of Allied Health Sciences, Augusta University, 1120, 15th St, Augusta, GA 30912, USA; 6 Department of Physical Therapy, College of Allied Health Sciences, Augusta University, 1120, 15th St, Augusta, GA 30912, USA

**Keywords:** Parkinson’s diseases, brain-gut axis, microbiome, intestinal barrier, permeability, leaky gut, niacin, butyrate, GPR109A, nutraceutical

## Abstract

Dysbiosis is implicated by many studies in the pathogenesis of Parkinson’s disease (PD). Advances in sequencing technology and computing have resulted in confounding data regarding pathogenic bacterial profiles in conditions such as PD. Changes in the microbiome with reductions in short-chain fatty acid (SCFA)-producing bacteria and increases in endotoxin-producing bacteria likely contribute to the pathogenesis of PD. GPR109A, a G-protein coupled receptor found on the surface of the intestinal epithelium and immune cells, plays a key role in controlling intestinal permeability and the inflammatory cascade. The absence of GPR109A receptors is associated with decreased concentration of tight junction proteins, leading to increased intestinal permeability and susceptibility to inflammation. In inflammatory states, butyrate acts via GPR109A to increase concentrations of tight junction proteins and improve intestinal permeability. Niacin deficiency is exacerbated in PD by dopaminergic medications. Niacin supplementation has been shown to shift macrophage polarization from pro-inflammatory to an anti-inflammatory profile. Niacin and butyrate, promising nutrients and unique ligands for the G protein-coupled receptor GPR109A, are reviewed in this paper in detail.

## 1. Introduction

The gut microbiome and its composition have been studied and implicated in the pathogenesis of multiple conditions, such as asthma, obesity, inflammatory bowel disease (IBD), autism spectrum disorders (ASD), and Parkinson’s disease (PD) [1,2,3,4,5,6]. The pathological bacterial species in the gut microbiome driving pathogenesis have not been identified in PD. As technology and computing power have improved over time, the cost of sequencing microbial samples has decreased, allowing for the characterization of dysbiosis in various conditions. Dysbiosis in ASD is the most studied. Gut microbiome characterization efforts in the ASD population, similar to PD, have encountered similar issues across studies such as recruitment of subjects in small, readily available cohorts, lack of sufficient control groups, and failure of accounting for confounding effects on the microbiome such as diet, geographic location, medications, surgeries, antibiotics, and probiotic use. Therefore, these studies demonstrate divergent results. Furthermore, chronic constipation, which is highly prevalent in both ASD and PD, may also alter the gut microbiome. Another consideration is whether the collection of specimens via stool or mucosal biopsy (biofilm sampling) is more reflective of brain–gut microbiome interactions. Shifts in microbiome composition obtained from ileal and cecal biopsies from ASD patients demonstrated correlations with lower mRNA levels of genes involved in carbohydrate digestion, increased serum serotonin, increased levels of inflammatory cytokines, and higher prevalence of co-existing functional gastrointestinal disorder (FGID) diagnoses meeting Rome III criteria [7,8].

Increased intestinal permeability caused by the loss of tight junction proteins in the setting of intestinal inflammation is the prevailing hypothesis for ‘leaky gut’ [9]. In line with the aforementioned phenomena, researchers are focused on different mechanisms by which the intestinal barrier can be compromised. Investigation of intestinal and immune cell receptors involved in intestinal permeability, influenced by short-chain fatty acids (SCFAs) and inflammatory markers, has provided researchers mechanistic insights [10,11,12,13,14,15]. SCFAs such as butyrate are ligands that promote intestinal epithelial cell stability, are sources of energy, and enhance the intestinal barrier [16].

Niacin, or Vitamin B3, is of growing interest as a potential agent for the amelioration of PD. In studies involving animal subjects, the vitamin also proved to have neuroprotective involvement in other neurological pathologies, such as traumatic brain injury and multiple sclerosis. Niacin has proven anti-inflammatory effects, suppressing pro-inflammatory gene expression on M1 macrophages and improving vascular permeability [17]. Both niacin and butyrate are ligands for the G-protein coupled receptor, GPR109A [12,17]. The exact pathway that intestinal inflammation leads to neuro-inflammation and neurodegeneration in PD remains unknown. In this paper, we aim to review the gut microbiome, intestinal barrier permeability, their relation to pathogenesis, and the potential therapeutic roles of butyrate and niacin in PD.

## 2. Parkinson’s Disease (PD) as Brain–Gut Disorder

PD is the second most common neurodegenerative disorder with debilitating motor and non-motor symptoms, affecting 1–2% of the population over the age of 65 [18]. The pathognomic change in PD is the accumulation of misfolded and aggregated α-synuclein, called Lewy bodies, in motoneurons, leading to loss of dopaminergic neurons in the substantia nigra [19]. Gastrointestinal (GI) symptoms are the predominant non-motor features and part of the widespread autonomic dysfunction in PD, which are observed in both early and late-stage disease [20]. PD patients more frequently experience drooling, dysphagia, dyspepsia, gastroparesis, bloating, and/or constipation with significant impairment in quality of life [21]. Constipation, the most common GI symptom, often precedes motor symptom onset by more than 15 years; increasing constipation severity corresponds to a 3.3–4.2 hazards ratio for subjects developing PD [22,23,24]. Chronic constipation in PD patients is a multifaceted brain-gut and neuromuscular disorder related to dysbiosis, efferent brain–anal axis neuropathy, visceral hypersensitivity, intestinal methanogen overgrowth, rectal hyposensitivity, and anorectal incoordination [25,26,27].

Lewy bodies are present in the enteric nervous system eight years prior to the central nervous system [28,29]. Abnormal activation of the intestinal immune response by microbial products and toxins crossing the intestinal barrier elicits neuropathological changes in the enteric nervous system (ENS). This cascade of events leads to an initial insult, for example, α-synuclein misfolding and LRRK-2 upregulation in PD, thereby developing GI dysfunctions featuring the early onset of neurodegenerative disease in the gut [30,31,32]. Braak postula suggests the progression of Lewy pathology from the gut to the brain via the vagal innervation of the stomach based on extensive autopsy examinations of PD patients [33]. The stomach was assumed to be a site of prolonged antigen exposure, and susceptible unmyelinated vagal neurons served as the most direct path in the brain–gut axis, which runs from the dorsal motor nucleus in the medulla and extends through the abdomen to the viscera [34]. A similar, retrograde prion-like progression of Lewy pathology could occur via the unmyelinated motoneurons of Onuf’s nucleus, which also send projections to the midbrain, innervating the rectum, another site of prolonged antigen exposure [24]. In addition, a disturbed gut-microbiome axis and altered metabolome, which include SCFA, branched-chain amino acids, and peptidoglycans, may also increase intestinal permeability, drive diffuse neuro-inflammation, and potentiate the pathogenesis in PD [35]. Regardless of the mechanism of progression, neuro-inflammation and subsequent neurodegeneration in PD are diffuse. Neuronal α-synuclein aggregates have been found in tissues throughout the body, including tissues outside the central nervous system, such as the skin, adrenal medulla, cardiac plexus, submandibular glands, and olfactory bulb [36].

## 3. Gut Microbiome

The emergent of data highlighting the importance of the gut microbiome in health and disease, specifically the well-established connection between PD and the gut, have raised the possibility that the dysbiosis of the gut microbiome may play a role in PD pathogenesis (Table 1). [37]. While characterizing the gut microbiome by taxonomy is important, it is the ratio between commensal and pathogenic bacteria that alters the integrity of the intestinal barrier [38]. Inflammatory cell-signaling markers have also been observed to change intestinal permeability, as well as lead to cerebrospinal fluid (CSF)/central nervous system (CNS) inflammation [39,40].

The brain–gut axis, bidirectional neural connections from the CNS to the ENS, is evolving to incorporate the gut microbiome. Investigation of the microbiome–gut–brain axis has led researchers to propose a GI origin pathogenesis for PD and other neurodegenerative disorders [44,45,46]. A recent microbiome-wide association study by Wallen et al. found three clusters of co-occurring microorganisms in PD with an overabundance of a polymicrobial cluster of opportunistic pathogens, reduced levels of SCFA-producing bacteria, and/or elevated levels of carbohydrate metabolizers commonly known as probiotics [47]. In a study of 24 PD patients compared to 14 healthy controls, proportions of endotoxin-producing bacteria, Actinobacteria and Proteobacteria, were increased, whereas populations of SCFA-producing bacteria, *Bacteroides*, *Prevotella*, and *Ruminoccoccus*, were decreased, as shown in Table 1 [41]. The presence of SCFA-producing bacteria ferments resistant non-starch polysaccharides, non-digestible oligosaccharides, and dietary fibers to produce SCFAs. SCFAs such as acetate, butyrate, propionate, formic acid, and isobutyric acid decrease intestinal inflammation and downstream pathology in various diseases [11,14,48].

## 4. Intestinal Barrier

**Figure 1:**
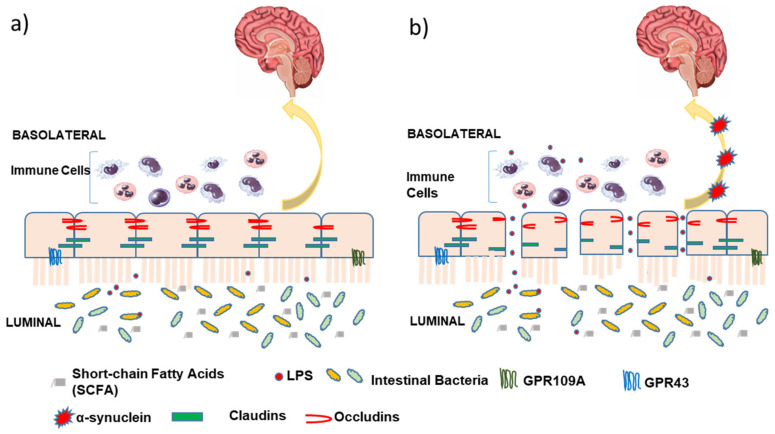
(**a**) Normal intestinal barrier with intact and functional tight junction proteins (Occludins/Claudins); (**b**) Leaky gut with increased intestinal permeability and dysfunctional tight junction proteins. Lipopolysaccharide (LPS) and bacterial toxins result in neuro-inflammation and Lewy pathology in PD pathology.

The intestinal barrier comprises a single layer of epithelial cells tightly stitched together by a special group of tight junction proteins such as claudins, occludens, tricellulin, and cadherins, shown in Figure 1. These tight junction proteins play an important role in maintaining gut permeability and homeostasis [49]. The luminal surface of the intestinal barrier is the largest body surface in close contact with external pathogens and the gut microbiome. Therefore, optimal functioning of these tight junction proteins is critical to the protection of the enteric nervous system, vasculature, and other crucial structural elements on the tissue side.

Gut microbiome and their bacterial byproducts can activate immune cells to regulate the expression of pro-inflammatory cytokines, such as TNF-α, IL-1β, and IL-6, which, in turn, act on tight junctions to increase barrier permeability [50]. These pro-inflammatory cytokines also initiate the recruitment of neutrophils, monocytes, and components from circulation to sites of inflammatory insult, thus prolonging the inflammatory response. Bacterial lipopolysaccharide (LPS) is a well-known endotoxin that is a potent trigger for TNF-α production [51]. GPR109A, a luminal G-protein coupled receptor on the intestinal epithelial surface, is a promising target for the modulation of intestinal permeability and potential reversal of leaky gut.

## 5. GPR109A

GPR109A, also known as HCAR2, is expressed on both hematopoietic and non-hematopoietic cells and serves as a receptor for both niacin and butyrate. GPR109A is found on dendritic cells and macrophages in addition to the intestinal epithelium. Downstream activation of this G-protein coupled receptor is involved in IL-8 and IL-10 production, which can influence regulatory T (Treg) cells to prevent or decrease inflammation [15,48]. The anti-inflammatory action of niacin occurs through a similar cascade of intramolecular events [52]. In a study led by Singh et al., Foxp3, a Treg cell marker and transcriptional regulator expression was decreased within the lamina propria of GPR109A-knockout mice compared to wild-type (WT) mice [15]. The anti-tumor molecular mechanism suggested by Singh et al. [15] includes a central role of GPR109A mediated anti-inflammatory response elicited in macrophages and dendritic cells, which in turn leads to differentiation of Tregs and IL-10 producing CD4+ T-cells. It has been shown that colonic health is highly dependent on the SCFAs such as butyrate, which are generated by gut microbiota by fermentation of dietary fibers. Changes in frequency of butyrate producing commensals is highly diminished in colonic diseases like ulcerative colitis and colon cancer [53,54]. It implies that the microbiome with or without GPR109A has a bidirectional relationship with intestinal inflammation and permeability.

Populations of Actinobacteria and Firmicutes increase, whereas Bacteroidetes decreases with age, resulting in an increased Firmicutes/Bacteroidetes (F/B) ratio [55]. High F/B ratios have also been observed in the setting of sepsis and are being studied to serve as a measure of sepsis severity [56]. Chen et al. found that GPR109A^−/−^ mice had increased Firmicutes, Verrucomicrobia, and Proteobacteria proportions when compared to WT mice [10]. The study also demonstrated a significant improvement in survival of GPR109A^−/−^ mice that underwent a fecal transplant of specimens from WT mice. This exemplifies the relationship between the composition of the microbiome, GPR109A presence, and the intestinal barrier’s integrity [10].

Chen et al. further investigated the colonic protective effect of GPR109A in the GPR109A^−/−^ mice [10]. The presence of GPR109A promotes survival in the setting of sepsis, as investigators demonstrated that GPR109A^−/−^ mice had a decreased survival rate, bodyweight loss, and increased disease severity. An increase in levels of pro-inflammatory IL-6 and IL-1β were seen in GPR109A^−/−^ mice compared to WT mice. GPR109A^−/−^ mice undergoing cecum ligation and puncture (CLP) to replicate sepsis were found to have decreased concentrations of tight junction proteins claudin-1, claudin-2, ZO1, ZO2, and occludin compared to WT [10].

## 6. Butyrate

Butyrate interacts with the GPR109A receptor and exhibits anti-inflammatory and anti-carcinogenic properties [12]. Butyrate is also the intestinal epithelial cells’ most-utilized SCFA for energy, making its investigation of the utmost importance for intestinal well-being [15]. In the study led by Feng et al., 24 piglets weaned at 21 days with diarrhea were divided into two groups. One group was fed with the basal diet and the other with basal diet plus 2000 mg/kg sodium butyrate. This experiment lasted a total of 21 days in which the diarrhea rate, frequency, and index were decreased compared to control. It was determined that intestinal permeability also was influenced by the addition of sodium butyrate, with upregulation of occludin and claudin-3 in the ileum and occludin, claudin-3, and ZO-1 expression in the colon. Using Western Blot analysis, ERK1/2, Akt, and P38 were phosphorylated, which indicated activation of the GPR109A pathway. Pro-inflammatory markers such as TNF-α were found to be significantly decreased with the administration of butyrate [12]. In another study, Yan et al. investigated the effects of butyrate on tight junction proteins on an LPS-induced inflammation model. They found that butyrate increased concentrations of claudins-3 and 4 and their mRNA expression in a dose-dependent manner, as well as prevented downregulation of Akt by LPS [57]. These studies suggest that butyrate may enhance tight junction expression through the Akt/mTOR pathway.

## 7. Niacin

PD patients are found to have significantly decreased niacin levels compared to age-matched healthy controls [52]. This has been attributed to both the disease itself and the medication used to attenuate motor symptoms [58]. Sinemet, the most commonly used PD medication, includes carbidopa, which prevents the conversion from l-dopa to dopamine within the peripheral nervous system. This allows more dopamine to be taken up by the central nervous system and used where it is scarce after dopaminergic cell loss. Unfortunately, a consequence of carbidopa is reduced conversion of tryptophan to niacin. Sinemet dose and frequency are increased over time as PD symptoms worsen, but concurrently side-effects also worsen.

**Figure 2:**
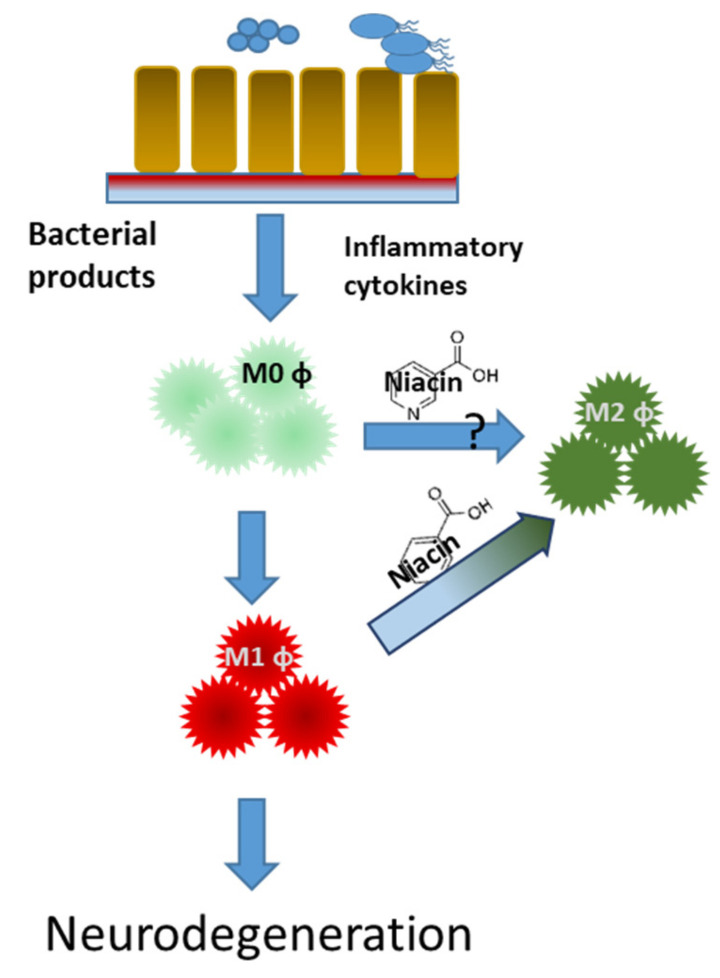
Niacin shifts macrophage polarization from pro-inflammatory to an anti-inflammatory profile in Parkinson’s disease patients. Naïve (M0 φ) pro-inflammatory (M1 φ) and anti-inflammatory (M2 φ) macrophages.

There are multiple forms of Vitamin B3, including nicotinic acid, nicotinamide, and nicotinamide riboside. Although all three forms provide a natural source of nicotinamide adenine dinucleotide (NAD) to the body, only nicotinic acid (niacin) binds to the anti-inflammatory GPR109A receptor [59]. In a clinical trial, it was found that the GPR109A receptor is upregulated in the white blood cells (WBCs) of PD patients [52]. After taking daily niacin supplements, the levels of the GPR109A receptor were reduced to a similar level of age-matched healthy controls. Subsequent anti-inflammatory effects have also been found in patients taking niacin. A shift in macrophages from a pro-inflammatory phenotype to an anti-inflammatory phenotype has been found after niacin supplementation, as shown in Figure 2 [60]. In LPS-activated mouse microglial cells, niacin was able to reduce IL-6 and IL-1β production [17]. Moreover, when GPR109A was downregulated with siRNA, niacin was not able to produce this effect.

A case report of a PD patient taking a high-dose niacin supplement for high triglyceride levels serendipitously found a reduction in motor symptoms, such as tremor and rigidity [61]. Unfortunately, large doses of niacin can result in flushing, secondary to the action on Langerhan’s skin cells, which have high levels of the GPR109A receptor. The PD patient taking a large dose of niacin daily had to quickly discontinue due to flushing and uncontrollable nightmares [61]. Fortunately, niacin doses of 500 mg or less, when taken with food and water, typically have little to no flushing effect. Moreover, flushing typically decreases with the continuation of niacin supplements. Other benefits to PD with niacin supplementation include an increase in quality of life [60,62] and motor symptoms [61,62]; fatigue and depression in PD may also benefit. Effects of niacin supplementation on mechanistic changes in pathophysiology, such as changes in pathologic α-synuclein levels, mitochondrial dysfunction, and leaky gut, should be investigated further.

## 8. Reactive Oxygen Species (ROS)

Oxidative stress, resulting from an imbalance in oxidants and antioxidants, and mitochondrial dysfunction are highlighted as a central feature of neurodegenerative diseases including PD, Alzheimer’s disease (AD), and Huntington’s disease (HD). Reactive oxygen species (ROS)-mediated oxidative DNA damage is one of the prominent features in PD. Multiple ROS biomarkers have been used to investigate the severity of these diseases [63,64]. Forsyth et al. showed that *E. coli* penetration into the intestinal mucosa was more frequent in PD patients compared to controls and the amount of enteric αSyn translocation correlated with increases in intestinal permeability and oxidative stress [65]. β-Hydroxybutyric acid (BHBA) acts on microglia to suppress LPS-induced inflammation through GPR109A to inhibit pro-inflammatory enzyme (iNOS and COX-2) and pro-inflammatory cytokine (TNF-α, IL-1β, and IL-6) production via the NF-κB signaling pathway in PD model rats [66]. Another study found that butyrate activated the AMPK signaling pathway through GPR109A to promote NRF2 nuclear accumulation and H3K9/14 acetylation, subsequently exerting antioxidant effects [67]. Above experiments show that butyrate and related compounds have important antioxidant function and can ease oxidative stress with synergistic effect on GPR109A signaling pathways, which may represent potential targets for therapeutic intervention to prevent or slow the progression of PD.

## 9. Other Nutrients

Other nutrients have been shown to be neuroprotective or beneficial for PD (Table 2). Omega-3 and vitamin E supplementation, taken together, were found to improve PD symptoms and decrease serum C-reactive protein (CRP) levels [13]. Vitamin D deficiency is also common in PD patients, although whether it is a primary deficiency or secondary has yet to be determined [68]. Vitamin D supplementation in vitro and animal models has demonstrated a decline in PD disease progression [69,70]; however, human studies have been controversial [71,72,73]. Anderson et al. investigated high consumption of vitamin D in humans, finding an increase in PD risk [73], while another human study showed vitamin D3 supplements to prevent progression of PD based on Hoehn and Yahr staging [72]. Interestingly, the vitamin D receptor is highly expressed in the substantia nigra, the area of the brain most affected in PD [74]. While vitamin C is neuroprotective [75], supplementation has been correlated with increased PD risk [76]. However, one study with supplementation of both vitamins C and E demonstrated a decrease in disease progression [77]. Clinical trials have also shown protective effects of vitamin E for PD patients [78], substantiated by animal studies showing protective effects of dopaminergic cells [79].

Many other nutrition-rich foods may be beneficial in PD, including fresh fruits, vegetables, coffee, and tea, which were all found to improve outcomes or decrease the risk of PD [92,93]. Further studies need to be performed to investigate which nutrients are the most important in PD, given that vitamin supplementation and dietary changes are safe, cheap, and easily accessible [94]. Finding benefits of vitamins and lifestyle changes in multifactorial diseases, such as PD, can lessen the need for increased dosage of dopaminergic medications and decrease disease progression. Such alternative treatment is needed as current medications only treat motor symptoms and lead to adverse effects, such as hallucinations and dyskinesias.

## 10. Conclusions

Microbiome studies in Parkinson’s disease, like other conditions, suffer from methodological variation and confounding factors and, therefore, specific bacteria involved in the pathogenesis of PD are difficult to characterize. However, an overall pattern corresponding to decreases in SCFA-producing bacteria and increases in endotoxin-producing bacteria have been observed in PD subjects. Fecal microbiome transplantation (FMT) and pre- and probiotics offer potential options for restoring the microbiome to PD patients. There are no robust data to adequately support FMT efficacy on motor and/or non-motor symptoms improvement or slowing the progression of PD or which route of administration and what content/volume of FMT is optimal. Therefore, we need more rigorous and well designed clinical trials to support FMT or the use of pro- and prebiotics in selected subgroups of PD patients in the future. GPR109A, a G-protein coupled receptor found on the surface of intestinal epithelium and macrophages, closely interacts with the microbiome to permit immune tolerance or trigger an inflammatory cascade. Loss of GPR109A is associated with decreased concentration of tight junction proteins and increased intestinal permeability. In inflammatory states, butyrate acts via GPR109A to increase concentrations of tight junction proteins and improve intestinal permeability. Niacin deficiency is exacerbated in PD by dopaminergic medications. Furthermore, niacin shifts macrophage polarization from pro-inflammatory to an anti-inflammatory profile. Future studies should study the effects of long-term supplementation of promising nutrients, such as butyrate and niacin, on their abilities to halt or reverse disease progression in PD. A deeper understanding of the GPR109A pathway in modulating intestinal permeability and its interplay in the microbiome–gut–brain axis may provide therapeutic options for multiple inflammatory and other neurodegenerative conditions.

## Figures and Tables

**Table 1:** Summary of significant changes in populations of bacterial taxa observed in gut microbiome studies of Parkinson’s disease (PD) patients.

Levels Compared to Controls	Bacteria Phylum/Family/Genus	References
Decreased	Bacterioidetes, Enterobacteriaceae, Enterococcaceae, Faecalibacterium, Lactobacillaceae, Lachnospiracea, Prevotellaceae, *Ruminococcus*, *Sediminibacterium*	[38,41,42,43]
Increased	Actinobacteria, *Akkermansia*, *Anaerotruncus*, *Aquabacterium*, *Bifidobacterium*, *Butryociococcus*, *Clostridium*, *Holdemania*, Proteobacteria, *Sphingomonas*	[38,41,42,43]

**Table 2:** List of nutrients that were tested in clinical trials with outcomes for PD.

Nutrient	Dose	*n* *	Function	Outcome	Ref.
Niacin	250 mg	46	support and stabilize mitochondrial function	improvement in Unified Parkinson’s Disease Rating Scale (UPDRS) III scores, improvement in cognition	[62]
	100 mg, 250 mg	46		changes in macrophage polarization, improved Quality of Life (QoL, reduction in rigidity and bradykinesia	[17,60,61]
Glutathione	1400 mg	21	Antioxidant, neuroprotection	UPDRS—Activities of Daily Living (ADL) + motor scores did not significantly change. Possibility of a mild symptomatic effect. The high-dose group showed improvement in total UPDRS and motor subscore over baseline	[80]
	300, 600 mg	30			
	100, 200 mg	41			
*N*-acetyl Cysteine	150 mg/Kg	6	Antioxidant, neuroprotection	Significantly increased dopamine transporter (DAT) binding in the caudate and putamen. UPDRS scores were significantly improved. Peripheral anti-oxidant measures increased significantly, but indicators of oxidative damage were unchanged	[81,82,83]
	6000 mg	8			
Hydrogen water	1000 mL	17		Significant improvement in total UPDRS scores. This study will confirm whether H2 water can improve PD symptoms	[80]
	1000 mL	178			
Creatinine minocycline	10 g/d 200 mg/d	67 66	support and stabilize mitochondrial function Antioxidant	creatinine group showed improvement in UPDRS3 scores after 18 months	[84]
Co-enzyme Q10	300 mg/d		Antioxidant, neuroprotection	no benefits at mid-stage PD	[85,86]
Lipoic Acid			Antioxidant, Anti-inflammatory, neuroprotection		[87]
Vitamin E and Omega-3 fatty acids	400 IU and 1000 mg flaxseed oil	30 treated 30 placebo	Antioxidant Antioxidant, Anti-inflammatory, neuroprotection		[88]
dephrenyl/ Vitamin E	10 mg/d 2000 IU		Antioxidant, Anti-inflammatory, neuroprotection	no benefits reported	[89,90]
Vitamin D3	NA	NA	Antioxidant, neuroprotection	Lowered serum levels of Vita D3 in PD patients. High Vit D levels are protective against PD	[91]

* *n*: number of subjects recruited into the study.
